# Molecular Investigation of *Plasmodium spp* From Faecal Samples of *Macaca fascicularis* in Indonesia

**DOI:** 10.1155/vmi/9331597

**Published:** 2026-08-31

**Authors:** Josephine Elizabeth Siregar, Firmanul Hasan, Normalita Eka Pravitasari, Andreas Yoga Aditama, Wanda Kuswanda, Lis Rosmanah, Uus Saepuloh, Huda Shalahudin Darusman, Fauzi Muh, Ita Margaretha Nainggolan, Syahputra Wibowo, I. Made Artika

**Affiliations:** ^1^ Eijkman Research Center for Molecular Biology, National Research and Innovation Agency, Jalan Raya Bogor Km. 46 Cibinong, Bogor 16911, Indonesia, brin.go.id; ^2^ Department of Biochemistry, Faculty of Mathematics and Natural Sciences, IPB University, Dramaga Campus, Bogor 16680, Indonesia, ipb.ac.id; ^3^ School of Tropical Medicine and Global Health, Nagasaki University, Nagasaki, Japan, nagasaki-u.ac.jp; ^4^ Ministry of Primary and Secondary Education, Jl. Jend. Sudirman Kompleks Kemendikbud, Jakarta 10270, Indonesia; ^5^ Research Center for Biota Systems, National Research and Innovation Agency, Jalan Raya Bogor Km. 46, Cibinong 16911, Indonesia, brin.go.id; ^6^ Primate Research Center, IPB University, Jalan Lodaya 2 No 5, Bogor 16151, Indonesia, ipb.ac.id; ^7^ Department of Epidemiology and Tropical Diseases, Faculty of Public Health, Universitas Diponegoro, Semarang 50275, Indonesia, undip.ac.id

**Keywords:** faecal samples, *Macaca fascicularis*, mitochondrial DNA, *Plasmodium* spp, simian malaria

## Abstract

Threats to Malaria Elimination Program (MEP) are arising, such as drug‐resistant parasites, insecticide‐resistant mosquitos and zoonotic malaria. In Southeast Asia, zoonotic emergence of macaque malaria, *Plasmodium knowlesi*, has been a major concern to the MEP. This parasite is recently established and potentially localised in wild populations of *Macaca fascicularis* (long‐tailed macaques), which are indigenous to South‐East Asia. Besides *P. knowlesi* infection, the macaque is also infected by *P. inui* and *P. cynomolgi*, which could be transmissible to humans. However, the parasitology data from Indonesian macaque populations are sparse. Noninvasive sampling methods are the primary tool for primate infectious disease ecology. The present study aimed to investigate *Plasmodium spp* from faecal samples collected (of long‐tailed macaques) in Indonesia. Faecal samples from semicaptive breeding sites and wild *M. fascicularis* have been collected. A total of 122 *M. fascicularis* faecal samples were collected from Java (West Java) and Sumatra (Lampung, Palembang and Jambi). DNA samples were extracted and subjected to nested polymerase chain reactions (nPCR) analysis using sets of mitochondrial DNA (mtDNA) primers to detect *Plasmodium* genus. There were 46 (37.7%) samples positive of malaria, and four species of *Plasmodium* parasites (*P. inui*, *P. cynomolgi*, *P. coatneyi* and *P. fieldi*) were detected in their natural host. None of the faecal samples were identified as being *P. knowlesi* positive. This study provides baseline information on the occurrence and diversity of simian malaria parasites in *M. fascicularis* populations in Indonesia using noninvasive samples and highlights the importance of further studies on simian malaria parasites in wildlife hosts.

## 1. Introduction

Malaria is a vector‐borne disease that remains a serious public health problem in many countries. Approximately half of the world’s population is at risk, mainly in tropical and subtropical areas [1, 2]. Malaria is caused by parasites of the genus *Plasmodium*, which are transmitted through the bites of infected mosquitoes. *Plasmodium* species are not restricted to human infections, and more than 200 species have evolved and coexist with other vertebrates including birds, reptiles and nonhuman primates [3]. *Plasmodium* species adapted to a particular vertebrate host species tend not to cross into other hosts, even when two hosts harbour very similar parasites [4].

There are five types of human malaria—*Plasmodium falciparum, P. vivax, P. malariae, P. ovale* and *P. knowlesi*—which differ in the periodicity of their life cycle as well as in disease outcomes. Among these, *P*. *knowlesi* is a public health threat in Southeast Asia. This parasite is primarily found in macaques, but it has been increasingly reported in humans over the past decade [1, 3].

A large number of human *P. knowlesi* cases were discovered in the Kapit District of Sarawak, Malaysian Borneo, during an investigation of presumed *P. malariae* infections, where patients presented with clinical symptoms and unusually high levels of parasitaemia [4]. Parasite DNA from malaria patients with atypical *P. malariae* infections was sequenced, and the results indicated *P. knowlesi* infection. *P*. *knowlesi*, which is morphologically similar to the human‐adapted *P. malariae,* is transmitted by mosquitoes of the *Anopheles leucosphyrus* group. The transmission is largely zoonotic and causes mild often self‐ limiting malaria with low parasite densities. Further research is needed to inform pre‐emptive control measures that may be required to prevent the full emergence of this parasite in human populations [4].

Macaque malaria in Indonesia has been reported previously [5]. However, studies on macaque malaria in the country remain limited [6]. This may be due to the difficulties associated with obtaining samples from nonhuman primates for malaria surveillance. It is almost impossible to collect blood samples from wild macaques because they are difficult to capture [6].

With improvement in molecular techniques, faecal samples have been used as a noninvasive approach to study hormones, genetics, disease and other biological parameters in rare or difficult‐to‐capture species. This approach has proven useful for the detection and molecular investigation of a wide range of pathogens, from viruses to helminths [7]. Several previous studies have successfully isolated and sequenced novel *Plasmodium* species from faecal samples of apes and Old World monkeys in Central Africa [8–10]. Faecal samples have also been employed to examine the diversity and zoonotic potential of ape *Plasmodium* parasites in different regions of Africa [11, 12]. The relative ease of faecal sample collection and its noninvasive nature make it an ideal tool for field studies, particularly when sampling large numbers of individuals.

Our laboratory has developed a molecular protocol to detect *Plasmodium* from faecal samples of *M. fascicularis* and *M. nemestrina*. The performance of this faecal PCR approach was previously evaluated using paired blood and faecal samples from naturally infected semicaptive macaques. The validated protocol showed comparable detection performance between faecal and blood samples, with a sensitivity of 96.7% (95% CI: 83.3%–99.4%) for active macaque malaria infections [13, 14]. Therefore, faecal DNA can be used as a reliable noninvasive template for molecular detection and species identification of Plasmodium in macaque samples.

The findings of this study provide molecular information on *Plasmodium* species infecting *M. fascicularis* populations in Indonesia and support future research on macaque malaria. This study was designed to investigate the occurrence, diversity and molecular characteristics of *Plasmodium* parasites using faecal DNA as a noninvasive sample source, rather than to estimate infection prevalence at the population level. The results provide additional insights into the genetic diversity of simian *Plasmodium* parasites in macaque hosts.

## 2. Materials and Methods

### 2.1. Sample Collection

Faecal samples of *M. fascicularis* were collected from semicaptive and wild macaque populations. A total of 122 faecal samples were included in this study, consisting of 90 samples from semicaptive macaques originating from West Java (*n* = 40); Lampung, Sumatra (*n* = 24); Palembang, South Sumatra (*n* = 26), and 32 samples from wild macaques originating from Jambi, Sumatra. Sampling was performed using a convenience sampling approach, where samples were collected based on the availability of macaque populations and accessible sampling sites during the study period.

Samples from semicaptive macaques were collected at the ex situ captive facility of the Primate Research Centre (PRC), IPB University, Dramaga Campus, Bogor, West Java, Indonesia (Lat −6.551418°, Long 106.725176°). Sampling was conducted at the end of the rainy season (March 2013). Semicaptive macaques were sedated for routine health screening procedures, during which faecal samples (approximately 200–300 mg) were collected.

Faecal samples from wild *M. fascicularis* populations were collected at Hutan Kota Muhammad Sabki, Jambi City, Indonesia (Lat −1.654537°, Long 103.585258°) during the early rainy season (September 2016). Sampling was performed by observing macaque groups leaving their sleeping sites in the morning for foraging activities. Shortly after leaving the sleeping sites, macaques commonly defecated, allowing the collection of fresh faecal materials after the animals moved away from the area. Each faecal sample was collected as an individual faecal bolus and identified based on spatial distance and differences in colour and texture. Dried, degraded, contaminated or mixed faecal materials were excluded to minimise the possibility of repeated sampling from the same individual. Whole faecal materials were collected beneath feeding trees where macaques had been active in the morning.

Faecal samples were placed into sterile 15‐mL centrifuge tubes containing RNAlater solution [15, 16] at a 1:1 ratio to preserve DNA integrity. Samples were stored at−20°C until DNA extraction.

### 2.2. Isolation of *Plasmodium* DNA

DNA from faecal samples was extracted using QIAamp DNA Stool Mini Kit (Qiagen) [7, 13]. Samples were lysed in ASL buffer overnight with vortexing to increase DNA yield; the rest of the protocol was followed up per manufacturer instructions. Filter pipette tips were used in all stages of processing to prevent cross‐contamination. The isolated DNA was aliquoted in several tubes and stored at −20°C for further analysis.

### 2.3. Detection of Malaria

Detection of *Plasmodium* utilised mitochondrial DNA primers for *Plasmodium* spp. that had been developed in our laboratory. Samples were first screened for malaria parasites using fragment of the mitochondrial small subunit ribosomal RNA (mt‐ssrRNA) *Plasmodium* primers and continued with primers targeting the cytochrome *b* (cyt*b*) gene for species‐specific detection Table 1. The PCR condition was adopted from Siregar et al. Several PCR conditions have been designed with the method of nested PCR and reamplification of parasite DNA [13, 17–19]. Four sets of primers with different targets were used in this study. The genes amplified were genotyped to further examine the species of *Plasmodium*.

**TABLE 1:** Primers for amplifying *Plasmodium* from faecal samples.

ID	Primer sets	Nest	Sequence	Product length (bp)	Gene	Reference for primers
Fragment mt‐ssrRNA	PfF 4595	reamp	GATTACAGCTCCCAGCAAC	424	Fragment mt‐ssRNA	[13]
	PfF 5019		GTTTAGCCAGGAAGTCAGCGTC			

Cyt*b*	PfF 4086	reamp	CCTTTAGGGTATGATACAGC	529	cytb	[13]
	PfR 4615		GTTTGCTTGGGAGCTGTAATC			

cyt*b*1	PfF 3368	nest1a	TGCCTAGACGTATTCCTGAT	734	cytb	[13]
	PfR4102		GCTGTATCATACCCTAAAG			

cyt*b*2	PfF 3368	nest2a	TGCCTAGACGTATTCCTGAT	349	cytb	[13]
	PfR3717		TATCTAAAACACCATCCACTCCA			

The pair of PfF4595/PfR5019 primers targeted a fragment of the mitochondrial small subunit RNA *Plasmodium* and utilised the reamplification method and targeted a 424‐bp fragment. The first and second PCRs were run using KAPA Taq DNA Polymerase (KAPA Biosystems, USA) under the following conditions: 5‐min denaturation at 94°C, 34 cycles of 15‐s denaturation at 94°C, 15‐s annealing at 56°C, 45‐s extension at 72°C and a final extension of 5 min at 72°C.

Three sets of primers were used to amplify the cyt*b* gene. The first set of primers was PfF 4086/PfR4615, also utilizing reamplification and targeting a 529‐bp fragment. Both PCRs were run using KAPA Taq DNA Polymerase (KAPA Biosystems, USA) under the following condition: 5‐min denaturation at 94°C, 34 cycles of 15‐s denaturation at 94°C, 15‐s annealing at 56°C, 1‐min extension at 72°C and a final extension of 5 min at 72°C.

The second and third primer pairs for cyt*b* gene were designed for nested PCR. The first nested primer set was PfF 3368/PfR 4102 and targeted a 734‐bp fragment, followed by a seminested reaction with the primer set PfF 3368/PfR 3717 utilizing the first product as template and targeting a 349‐bp fragment. Both PCRs were run using KAPA Taq DNA Polymerase (KAPA Biosystems, USA). The first nested PCR was run under the following conditions; 5‐min denaturation at 94°C, 34 cycles of 15‐s denaturation at 94°C, 15‐s annealing at 52°C, 45‐s extension at 72°C and a final extension of 5 min at 72°C. The second nested PCR was run under the same conditions with the first nested PCR but with an annealing temperature of 50°C. The first nested PCR was run with 2 μL of template, while the second PCR was run with 1 μL of product from the first round PCR.

All PCRs were run with a 25‐μL PCR mixture: 1‐2 μL of template, 2.5 μL of 10× KAPA Taq PCR buffer (standard Tris‐ammonium sulphate‐based buffers containing 15 mM MgCl_2_; Kapa Biosystems, USA), 10 pmol of each primer, 100 μM of dNTPs (Promega), 0.5 U of Taq DNA Polymerase (Kapa Biosystems, USA). All PCRs were run alongside negative and positive controls. To minimise the risk of cross‐contamination, DNA extraction, PCR master mix preparation, template addition and post‐PCR analysis were performed in separate laboratory areas using dedicated equipment and sterile filter tips. Extraction blanks and no‐template controls were included in each PCR run to monitor possible contamination. Positive controls were handled separately and added after all samples had been prepared. PCR runs showing amplification in negative controls were considered invalid and repeated.

### 2.4. Direct Sequencing of PCR Product

The amplified products were purified using a QIAamp PCR purification kit (QIAGEN, Germany) and directly sequenced using Forward and Reverse primer for PCR cyt*b* and fragment of the mitochondrial small subunit ribosomal RNA (mt‐ssrRNA) *Plasmodium* in an Applied Biosystems 3130/3130XL Genetic Analyser (Applied Biosystems).

### 2.5. Sequence Analysis

Sequences were determined directly using sequencing primers. Electropherogram files obtained from Sanger sequencing were manually inspected to evaluate sequence quality and to assess the presence of well‐defined overlapping peaks (double peaks) at specific nucleotide positions, which indicated mixed‐species infections. To resolve and justify the base identities, the ambiguous positions were manually verified and verified against reference sequences of sympatric simian malaria parasites. The sequences obtained were BLAST to the reference sequences and aligned using ClustalW program and BioEdit program (Ibis Biosciences, CA, USA) to determine the identity of the *Plasmodium* species. The evolutionary distances were estimated using the MEGA program. A phylogenetic tree was constructed by the neighbour‐joining (NJ) method.

## 3. Results

### 3.1. Detection of Malaria Parasite

In this study, 122 *M. fascicularis* faecal samples were screened for *Plasmodium* malaria using nested PCR and reamplification of DNA parasites.

The overall detection rate of malaria infection from all samples was 37.7% (46/122) by PCR detection using fragment of the mitochondrial small subunit ribosomal RNA (mt‐ssrRNA). The *Plasmodium* detection rate from West Java, Lampung, Palembang and Jambi samples was 27.5%, 29.2%, 73.1% and 28.1%, respectively; the Palembang samples showed the highest *Plasmodium* detection rate (73.1%). The identification of positive samples was done by BLAST analysis to the reference sequences. We identified four species of *Plasmodium* from all positive samples, being *P. inui, P. cynomolgi, P. coatneyi* and *P. fieldi* Table 2.

**TABLE 2:** Malaria infection in *M. fascicularis*.

Sample no	Origin	Total samples	Infection rate (%)	Primer use for sequencing	Identification sequences by BLAST analysis (samples)
1	Semicaptive breeding PRC IPB University (originated West Java)	40	27.5	PfF 4086 and PfF 3368	*P. inui* (8),
				PfF 4595	*P. cynomolgi* (2)
				PfF 4595	*P. coatneyi/P. inui* (1)
2	Semicaptive breeding PRC IPB University (originated Lampung, Sumatra)	24	29.2	PfF 4086 and PfF 3368	*P. inui* (6),
				PfF 4595	*P. fieldi* (1)
3	Semicaptive breeding PRC IPB University (originated Palembang, Sumatra)	26	73.1	PfF 4086 and PfF 3368	*P. inui* (19)
4	Wild macaques (originated Jambi, Sumatra)	32	28.1	PfF 4595	*P. inui* (9)
	Total samples	122			46

*P. inui* is the dominant *Plasmodium* species found from all sample sites. All samples from Palembang (19 samples) and Jambi (9 samples) have mono‐infection of *P. inui*, and no other *Plasmodium* species were detected in those Provinces. As for samples from West Java, there are three *Plasmodium* species identified; single infection of *P. inui* (8 samples), single infection of *P. cynomolgi* (2 samples) and mixed infection of *P. coatneyi/P. inui* (1 sample). Two *Plasmodium* species were identified from Lampung, six samples with mono‐infection of *P. inui* and one sample with mono‐infection of *P. fieldi*. The detection rate of mono‐infection from total samples was higher (97.8%) than that of mixed infection (2.2%). Only one macaque from West Java has mixed infection of *P. coatneyi/P. inui*.

### 3.2. DNA Sequence Analysis of *Plasmodium* spp

Since the majority of positive samples were identified as *P. inui*, we undertook further analysis of sequence variation of the cyt*b* gene. The sequence of cyt*b* gene (around 500 bp) from all *P. inui* samples was aligned by the ClustalW program. No variation was found in partial cyt*b* sequence among *P. inui* samples from West Java, Lampung and Palembang. As for samples from Jambi, the cyt*b* gene amplification was still difficult to obtain. One possible reason was DNA from faecal samples might have been degraded before DNA extraction process. Therefore, we repeated the next amplification using shorter primer pairs. Out of the samples analysed, three samples were selected (each from West Java, Lampung and Palembang) for further sequencing of the full length of cyt*b* gene (1.131 bp) and do phylogenetic analysis with reference sequences.

In further analysis, cyt*b* gene sequence from samples West Java, Lampung and Palembang were aligned together with 10 Reference sequences from GenBank with accession number AB444115 (Perlis State, Malaysia), KJ569840 (Sabah, Malaysia), AB444110 (Kuala Selangor, Malaysia), KJ569848 (Sabah, Malaysia), KJ569849 (Sabah, Malaysia), KJ569850 (Sabah, Malaysia), GQ355483 (Taiwan), HM032052 (China), AB354572 (ATCC 30156 Laboratory Culture from West Malaysia) and AB444112 (India/Malaysia). We found that there was some polymorphism in cyt*b* gene among isolates of *P. inui* from the alignment results with the reference sequences. Based on those polymorphic samples, we have generated haplotype group of *P. inui* isolates Table 3.

**TABLE 3:** Haplotype sequence cytochrome *b* among *P. inui* isolates.

Strain	Base position																																									
	22	26	31	42	45	66	84	107	130	162	174	177	337	354	358	378	456	498	514	535	582	591	607	618	747	757	759	766	774	792	810	896	915	938	948	949	988	1051	1059	1094	1115	1116
AB354572.1 (Perak, Malaysia	T	C	G	G	T	T	T	T	A	A	T	T	G	T	G	T	T	T	T	G	C	T	G	T	A	G	A	A	T	T	A	A	G	G	T	C	T	A	T	G	C	T
AB444115.1 (Perlis, Malaysia)	T	C	G	G	T	T	C	T	A	A	T	T	G	T	G	T	T	T	T	G	C	T	G	T	A	G	A	A	T	T	A	A	G	G	T	C	T	A	T	G	C	T
AB444112.1 (Malaysia)	T	C	G	A	T	T	C	T	G	A	T	T	G	T	G	T	T	T	T	G	C	T	G	T	T	G	A	A	T	T	A	G	A	G	A	G	C	A	T	G	C	C
KJ569848.1 (Sabah, Malaysia)	T	C	A	A	C	A	C	T	G	A	C	T	G	T	G	T	T	T	C	G	T	A	G	A	T	A	T	G	C	T	G	G	A	G	A	G	T	A	C	A	C	C
KJ569849.1 (Sabah, Malaysia)	T	C	A	A	C	A	C	C	G	A	C	T	G	T	G	T	T	T	C	G	T	A	G	A	T	A	T	G	C	T	G	G	A	G	A	G	T	A	C	A	C	C
KJ569850.1 (Sabah, Malaysia)	T	C	A	A	C	A	C	T	G	A	C	T	G	T	G	T	T	T	C	G	T	A	G	A	T	A	T	G	C	T	G	G	A	G	A	G	T	A	C	A	C	C
HM032052.1 (China)	T	C	G	G	T	A	C	T	A	G	T	T	G	A	A	C	T	A	T	G	C	T	G	T	A	G	A	A	T	T	A	A	G	G	T	C	T	T	T	G	T	C
GQ355483.1 (Taiwan)	T	C	G	G	T	A	C	T	A	G	T	T	G	A	A	C	T	T	T	A	C	T	A	T	A	G	A	A	T	T	A	A	G	G	T	C	T	T	T	G	T	C
KJ569840.1 (Sabah Malaysia)	T	C	A	A	C	A	C	T	G	A	C	T	G	T	G	T	T	T	C	G	T	A	G	A	T	A	T	G	C	T	G	G	A	G	A	G	T	A	C	A	C	C
#26 (Lampung)	C	T	G	A	T	T	C	T	G	A	T	C	A	T	G	T	T	T	T	G	C	T	G	T	T	G	A	A	C	T	T	G	G	A	A	G	T	A	T	G	C	C
T3853 (West Java)	C	T	G	A	T	T	C	T	G	A	T	C	A	T	G	T	T	T	T	G	C	T	G	T	T	G	A	A	C	T	T	G	G	A	A	G	T	A	T	G	C	C
H07 (Palembang)	C	T	G	A	T	T	C	T	G	A	T	C	A	T	G	T	T	T	T	G	C	T	G	T	T	G	A	A	C	T	T	G	G	A	A	G	T	A	T	G	C	C
AB444110.1 (K. Selangor Malaysia)	T	C	G	C	T	A	C	T	G	A	T	T	G	T	G	T	A	T	T	A	C	A	G	A	T	G	T	A	T	C	G	G	A	G	T	G	T	A	C	G	C	C

*Note:* Sample codes of AB354572, AB444115, AB444112, KJ569848, KJ569849, KJ569850, HM 032052, GQ355483, KJ569840 and AB444110 are from GenBank (National Library of Medicine), while #26, T3853 and H07 are our samples.

From the haplotype sequences generated from the full sequence cyt*b* (Table 3), we analyse the geographical spread of *P*. *inui* and observe the divergence among them (Table 4). Our samples represented by sample #26 (Lampung), T3853 (West Java) and H07 (Palembang) are closely related to Reference sequence AB444112 (India/Malaysia) with divergence 0.8%, with *P. inui* isolates with reference number HM032052 (China) and GQ355483 (Taiwan) having the more variation compared to our isolates in this research with divergence of 1.9% and 2.0%. The isolates with most variation with divergence of ≥ 2.5% are shown by *P. inui* isolate reference numbers HM032052 (China) and GQ355483 (Taiwan) compared to all isolates from Sabah, Malaysia (KJ569848, KJ569849 and KJ569850).

**TABLE 4:** Mitochondrial (mt) sequence divergence among *P. inui* isolates.

Sample code	AB354572.1	AB444115.1	AB444112.1	KJ569848.1	KJ569849.1	KJ569850.1	HM032052.1	GQ355483.1	KJ569840.1	AB444110.1	#26	H.07	T3853
AB354572.1													
AB444115.1	0.001												
AB444112.1	0.009	0.008											
KJ569848.1	0.021	0.020	0.014										
KJ569849.1	0.022	0.021	0.015	0.001									
KJ569850.1	0.021	0.020	0.014	0.000	0.001								
HM032052.1	0.009	0.008	0.014	0.025	0.026	0.025							
GQ355483.1	0.010	0.009	0.015	0.026	0.027	0.026	0.003						
KJ569840.1	0.021	0.020	0.014	0.000	0.001	0.000	0.025	0.026					
AB444110.1	0.015	0.014	0.011	0.012	0.013	0.012	0.019	0.018	0.012				
#26	0.013	0.012	0.008	0.018	0.019	0.018	0.019	0.020	0.018	0.016			
H.07	0.013	0.012	0.008	0.018	0.019	0.018	0.019	0.020	0.018	0.016	0.000		
T3853	0.013	0.012	0.008	0.018	0.019	0.018	0.019	0.020	0.018	0.016	0.000	0.000	

**FIGURE 1:**
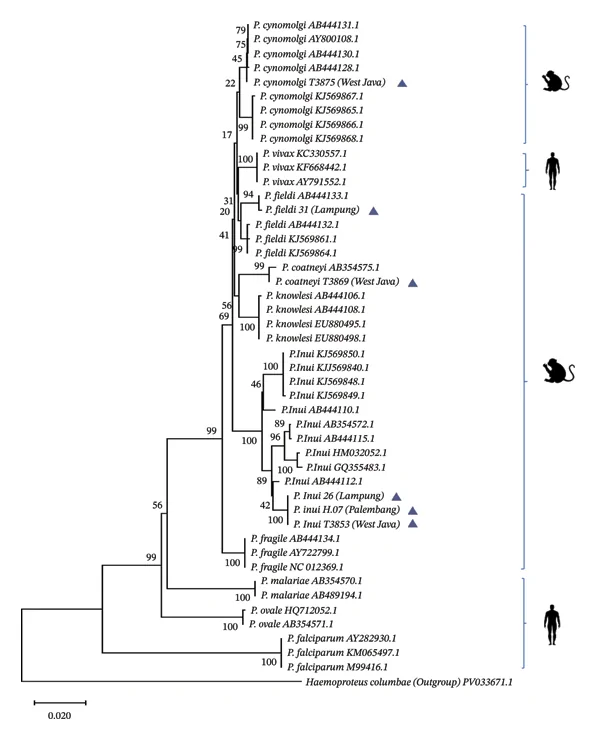
Phylogenetic tree of human and NHP malaria parasites. Notes: 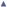 = our samples.

The sequences of cyt*b* gene of *P. inui* isolates collected from our research and from reference isolates were used to elucidate how they are clustered. To do this, a phylogenetic tree was constructed using the NJ method implemented in MEGA v.5 computer program. We also included some reference sequences of Human malaria (*P. falciparum*, *P. vivax*, *P. malariae* and *P. ovale*) and nonhuman primate malaria (*P. knowlesi*, *P. cynomolgi*, *P. coatneyi*, *P. fieldi* and *P. fragile*). The length of sequences of the cyt*b* gene is the same as those of our isolates. The NJ phylogenetic tree (Figure 1) shows the distribution of *P. inui* and other malaria parasites based on cyt*b* gene sequences.

## 4. Discussion

The high detection rate of *P. inui*–positive samples was observed among long‐tailed macaques from Palembang and Jambi, followed by West Java and Lampung. These detection rates represent the proportion of positive samples among the analysed samples at each sampling location and should not be interpreted as estimates of regional prevalence. Because samples were collected using a convenience sampling approach and sample sizes varied among locations, the sampled macaques may not fully represent the broader macaque populations in each province. A study conducted in Kapit, Sarawak, reported a similar pattern, where *P. inui* was the predominant *Plasmodium* species detected in long‐tailed macaques (82%) compared with other *Plasmodium* species [20]. In Sabah, Malaysian Borneo, it was also found that *P. inui* was the most common parasite in both *M. fascicularis* and *M. nemestrina* [21]. In addition, natural infections of *P. inui* in humans have been reported in Malaysia [22] and Thailand [23]. These findings highlight the importance of continued molecular monitoring of *Plasmodium* parasites in macaque reservoir hosts. However, the present study was not designed to evaluate zoonotic transmission risk, as no human samples, mosquito vector data or ecological interaction analyses were included. Therefore, the detection of *P. inui* in macaques does not directly demonstrate active transmission or spillover potential to humans. Comprehensive evaluation of zoonotic risk requires additional evidence integrating human infection data, vector surveillance and assessments of human–primate–vector interactions.

The infection of *P. cynomolgi* attracted in‐depth attention as several cases of natural infection in humans have been reported in different areas of Southeast Asian countries, such as Hulu Terengganu [24] and Sabah [25] in Malaysia, Pailin and Battambang in Cambodia [26] and Tak, Chanthaburi, Ubon Ratchathani, Yala and Narathiwat in Thailand [27]. It is important to note, however, that a recent retrospective study in Sabah, Malaysia, involving more than 2000 malaria samples from patients with symptomatic *Plasmodium* species infections, did not find *P. cynomolgi* infections [28]. Although previous reports indicate that *P. cynomolgi* can infect humans, detection of this parasite in macaques alone does not establish zoonotic transmission. We have investigated *P. cynomolgi* from West Java and Lampung, Sumatra, in macaque monkeys and found no interhost crossover (Figure 1).

*P. inui* isolates from our research are clustered together, and they have close geographic proximity to isolates from Peninsular Malaysia rather than the isolates from Sabah. This also happened with *P. inui* isolates from China and Taiwan, which are clustered separately, but they are also more closely related to our isolates compared to all isolates from Sabah.

Our *P. cynomolgi* isolate T3875 (West Java) is also closely related to *P. cynomolgi* isolates from Peninsular Malaysia, compared to the one from Sabah, Malaysia. This evidence is also the case with *P. fieldi* isolates. The Indonesian *P. inui* isolates analysed in this study were clustered together and showed identical in genetic variation based on the full‐length cyt*b* sequences. However, the current dataset does not provide sufficient evidence to conclude that *P. inui* represents a single population distribution in Indonesia. Although full‐length cyt*b* sequences were analysed, the limited number of samples and geographic coverage require cautious interpretation. Additional sampling from broader regions and the use of additional genetic markers will be required to further evaluate the population structure of *P. inui* in Indonesia.

Although direct Sanger sequencing is suitable for species identification when a dominant parasite sequence is present, this approach has limited sensitivity for detecting minority variants in mixed‐species or mixed‐clone infections. Therefore, low‐abundance mixed infections cannot be completely excluded in the present study. Future studies using more sensitive approaches, such as deep sequencing or cloning‐based methods, may provide additional resolution for detecting mixed infections.

In this study, we have not yet found any *P. knowlesi* isolated from faecal samples of Indonesian macaques collected. Further research is needed in the area where the macaques are naturally distributed, especially where there is intensive habitat sharing between humans and nonhuman primates (for example, due to deforestation). Despite infection of *P. knowlesi* in humans in Aceh Besar District (in the northern part of Sumatra) was reported [29], there are currently no published report of *P. knowlesi* cases in macaque in that specific area. This research highlights the importance of continued molecular monitoring of wild macaque populations as reservoir hosts to improve understanding of *Plasmodium* diversity and distribution in the Indonesian region.

## 5. Conclusion

This study identified four species of *Plasmodium* (*P. inui, P. cynomolgi, P. coatneyi* and *P. fieldi*) from *M. fascicularis* faecal samples collected from West Java, Lampung, Palembang and Jambi. *P. inui* is the dominant species at all sample sites, and this species is the most widely distributed monkey malaria parasite in the Asian region. All *P. inui* collected from our study have no polymorphism in full cyt*b* gene, and they all fall within the same population distribution.

## 6. Future Research

Further investigations of *Plasmodium* spp. from faecal samples of nonhuman primates are needed in other regions of Indonesia where macaques are naturally distributed. Broader sampling across different geographic areas will improve understanding of the distribution and genetic diversity of simian *Plasmodium* parasites. Future studies integrating molecular surveillance, vector data and human infection data are required to better evaluate the potential zoonotic risk of simian malaria parasites.

## Author Contributions

Josephine Elizabeth Siregar: conceptualisation, sampling, experimental work, molecular analysis, and manuscript drafting and editing.

Firmanul Hasan: experimental work, molecular analysis, and manuscript review and editing.

Normalita Eka Pravitasari: sampling, experimental work, molecular analysis, and manuscript review and editing.

Andreas Yoga Aditama: sampling, experimental work, molecular analysis, and manuscript review and editing.

Wanda Kuswanda: sampling and manuscript review and editing.

Lis Rosmanah: sampling and manuscript review and editing.

Uus Saepuloh: sampling and manuscript review and editing.

Huda Shalahudin Darusman: sampling and manuscript review and editing.

Fauzi Muh: molecular analysis and manuscript review and editing.

Ita Margaretha Nainggolan: molecular analysis and manuscript review and editing.

Syahputra Wibowo: molecular analysis and manuscript review and editing.

I. Made Artika: molecular analysis and manuscript drafting and editing.

## Funding

This initial research was supported by Toray Research Grant 2015 (No. 003/I/ITSF/SEK/2015) and the continuation of research was funded by the Research Organization for Life Sciences and Environment, National Research and Innovation Agency 2024 (NOMOR B‐1539/III.5/PR.03.06/5/2024) and 2025 (NOMOR 3/III.5/HK/2025).

## Disclosure

All authors have read and approved the manuscript.

## Ethics Statement

All procedures performed in studies involving animals were in accordance with the ethical standards of the Animal Care and Use Ethics Committee, National Research and Innovation Agency, reference number: 004/KE.02/SK/1/2025 and Primate Research Center, IPB University, reference number: IPB PRC–15–A008.

## Conflicts of Interest

The authors declare no conflicts of interest.

## Data Availability

The data that support the findings of this study are available from the corresponding author upon reasonable request.
